# Immune system and melanoma biology: a balance between immunosurveillance and immune escape

**DOI:** 10.18632/oncotarget.22190

**Published:** 2017-10-31

**Authors:** Anna Passarelli, Francesco Mannavola, Luigia Stefania Stucci, Marco Tucci, Francesco Silvestris

**Affiliations:** ^1^ Department of Biomedical Sciences and Human Oncology, University of Bari ‘Aldo Moro’, Bari, Italy

**Keywords:** melanoma, immune system, immunogenicity, immunoediting, immune escape

## Abstract

Melanoma is one of the most immunogenic tumors and its relationship with host immune system is currently under investigation. Many immunomodulatory mechanisms, favoring melanomagenesis and progression, have been described to interfere with the disablement of melanoma recognition and attack by immune cells resulting in immune resistance and immunosuppression. This knowledge produced therapeutic advantages, such as immunotherapy, aiming to overcome the immune evasion.

Here, we review the current advances in cancer immunoediting and focus on melanoma immunology, which involves a dynamic interplay between melanoma and immune system, as well as on effects of “targeted therapies” on tumor microenvironment for combination strategies.

## INTRODUCTION

Cutaneous melanoma (CM) is the most common skin cancer with an incidence that is rapidly increased in the past decade [1]. It is estimated that in 2017 there will be approximately 87,000 new cases of melanoma in USA and that 9,000 people will die of this cancer. Despite the encouraging clinical results of novel therapies, the prognosis is poor and partly dependent on the physical patterns of the primary lesion including thickness, involvement of lymph nodes and propensity of malignant cells to colonize distant tissues. The interplay of melanoma cells with other cells resident within the tumor microenvironment significantly influences the tumor biology as proliferation, differentiation and progression [2]. On the other hand, melanoma cells bear a peculiar immunogenic profile and provide a suitable model to investigate the molecular crosstalk of cancer cells with cells of the immune system and advances in this contest have discovered novel molecular targets on immune cells to develop efficient therapeutic strategies.

During the melanomagenesis [3], both cell proliferation and apoptosis are associated to the immune editing that includes inter-connected phases as *elimination* of tumor cells based on immunosurveillance, equilibrium between tumor and immune cells and *escape* or immune evasion. The e*limination* is based on sequential events leading to the anti-melanoma cytotoxicity by natural killer (NK) and dendritic cells (DCs), T-cells, B-cells either within the tumor microenvironment or in peripheral tissues [4]. The equilibrium consists in a prolonged phase during which the tumor cells are constantly suppressed whereas the next step is characterized by the selection of resistant variants that critically induce the cancer cell immunogenicity. This is the longest of the three phases required for immunoediting although its definite development may require over a period of years [5]. The equilibrium phase, therefore, is mostly characterized by a ‘quiescence’ during which either proliferation or expansion of cancer cells are counterbalanced by the adaptive immune system. In this context, T-cells are major players enrolled in activating the equilibrium phase, although tumor cell variants progressively lose major histocompatibility complex (MHC) class-I and -II molecules, thus releasing relevant amounts of antigens. Although a number of studies have demonstrated the role of IFN-γ in supporting the immunosurveillance, recent evidence clearly shows that lymphocytes are pivotal for this function. Furthermore, mouse models knockout for the recombination activating gene (*Rag)-*1 or *Rag*-2 fail to rearrange the lymphocyte antigen receptors, thus resulting in defective production of mature peripheral B cells as well as NK T cells (NKT) or αβ and γδ T cells. Moreover, Rag-2^−^/^−^ mice subcutaneously injected with the chemical carcinogen 3′-methylcholanthrene (MCA) showed a high incidence of sarcomas with respect to wild-type controls [6], while further studies emphasized the role of T cell subsets in the maintenance of immunosurveillance based on the increase susceptibility to the MCA-induced cancer of both TCR β^−/−^ and TCR δ^−/−^ mice. In addition, the primary role of αβ and γδ T cell subsets has been also demonstrated in promoting functional antitumor immune response [7] and IFN-γ production within tumor microenvironment. By contrast, the immune escape depends on the exhaustion of the immunosurveillance as well as on the activation of events that disable the immune-mediated recognition of malignant cells.

The functional exhaustion of the immune system depends on the persistent antigen exposition by melanoma cells and induces the hyper activation of inhibitory checkpoints on immune cells as result of a negative feedback for the cytotoxic T-cells [8, 9]. Similarly, the enrichment of the melanoma environment in tumor-associated macrophages (TAMs), regulatory T cells (Treg) and myeloid-derived suppressor cells (MDSCs) represents an additional mechanism that promotes the defective cytotoxicity of T-cells. On the other hand, the inefficient killing of malignant cells is mostly due to a direct effect of melanoma cells through the over-production of negative modulators of immune cell [10] including adenosine, tumor necrosis factor-β (TGF-β), vascular endothelial growth factor (VEGF) and indolamine 2,3-dioxygenase (IDO) [11, 12] as well as by the loss of both class I and II antigens of the major histocompatibility complex (MHC).

Based on the knowledge of the melanoma biology, new therapeutic strategies including CTLA-4, PD-1 and PD-L1/2 blockers, aimed at restraining the molecular interplay between tumor cells and effector immune cells, have been developed and results from extended clinical trials describe a revolutionary improvement in the management of melanoma patients [13–18].

Here, we revisit both cellular and molecular events that balance immunosurveillance and editing in melanoma biology as major mechanisms involved in tumor progression and a brief description of the innovative therapeutic strategies against this tumor.

### The immunogenic behavior of melanoma cells

Immunogenicity of a tumor is the capacity to induce adaptive immune responses that can prevent its growth and melanoma cells are considered highly immunogenic or capable of activating or modulating the adaptive immune response resulting in the balance between survival and proliferation [6]. However, despite this interpretation, the exact understanding of this process is still evolving. Different mechanisms regulate both antigen expression and presentation that thus appear as major events determining the tumor immunogenicity [19]. Notwithstanding the continuous crosstalk of melanoma cells with DCs and T-cells in both peripheral blood and lymph nodes, the immune editing during the progression of the disease is progressively lost. This event perhaps represents the major reason of the uncontrolled proliferation of tumor cells as well as of their migration capability to distant sites. On the other hand, the spontaneous tumor regression frequently observed in clinical practice is associated with functional T-cell activation and provides a potential explanation for the break between tumor proliferation and immune system control [20].

Generally, the immunogenicity has been considered for long time the boundary to discriminate self from non-self. Therefore, the immunogenicity requires that melanoma cells express adequate levels of antigens capable to elicit immune activation instead of immune tolerance. This, however, depends not only on the antigenicity of cancer cells but also on functional interactions among immune cells as well as on immunomodulatory factors released by the tumor cells [21].

The reasons underlying the immunogenicity [22] of melanoma cells are unclear and depend on molecular events that enhance their proliferation and expansion within the tumor microenvironment in the presence of inhibitory signals by interleukin (IL)-6, IL-10 and TILs, namely the tumor infiltrating lymphocytes. Their persistent activity against the malignant cells promotes the exposition of tumor neo-antigens deriving from continuous mutations that drive a consistent number of amino acid coding sequences as non-synonymous somatic mutations.

The molecular mechanisms that regulate the immune response in melanoma and its spontaneous regression sporadically observed in the clinical setting are unclear and are partially explained on the number of somatic mutations of coding exons and/or the splice junctions that recur in melanoma cells as well as on the high mutational load and the mutagenic signature induced by ultraviolet light [23]. Additional studies also showed that melanoma cells may acquire up to more then one hundred mutations per megabase thus bearing a high mutational load with respect to other malignant populations [24] and these mutations contribute to the generation of novel epitopes [25, 26].

The most relevant antigens expressed by melanoma cells include: i) differentiation antigens or tumor-associated antigens (TAAs) expressed by both normal and malignant melanocytes; ii) cancer/testis antigens [27] that are typical of many tumor types and germ line cells derived from normal adult testis; iii) tumor derived neo-antigens resulting from non-synonymous somatic mutations that are altered in their amino-acid coding sequence. The presence of TAAs in normal cells drive both central and peripheral tolerance for the selection of specific T-cells bearing a T-cell receptor (TCR) with low affinity for the antigen. This explains the tolerogenic effect to self-proteins observed in a few clinical trials using vaccines engineered with these antigens.

Therefore, the neo-antigens are considered ideal targets for immunotherapy and their detection could be a good biomarker to predict the efficiency of immune checkpoint inhibitors including both anti-PD-1 (programmed death-1) and anti-CTLA4 monoclonal antibodies (MoAbs) [28, 29]. In this context, recent studies demonstrated that metastatic patients with disease at high mutational load undergo long-term clinical benefit after CTLA-4 inhibition in a fashion almost similar to the effect of PD-1 inhibition in patients with colorectal cancer bearing defective mismatch repair proteins [30]. However, the mechanisms by which CTLA-4 inhibition boosts tumor-specific T cell activity are unclear but the efficacy of CTLA-4 blockade is correlated with the emergence of new T-cells primed against neo-antigens [31]. This could at least explain the modest cytotoxicity exerted by pre-existing T-cells observed during ipilimumab therapy as well as the relationship between neo-antigens and improvement of progression free survival (PFS) in melanoma patients treated with the anti-CTLA4 MoAb.

### Activation of the immune system in melanoma

A number of studies proved the relationship between defective immune system activity and melanoma cell proliferation, while others demonstrated that the variability of the antigenic repertoire is a critical factor for the immunosurveillance and melanoma progression [3, 32].

An efficient anti-melanoma immune response requires a fast and not-specific phase that activates the innate immunity before a specific adaptive stimulation of the immune system. Both phases induce apoptosis of melanoma cells through T-cell-mediated cytotoxicity and the efficiency of both T-cells and signals driven by TCR are thus central to counterbalance the melanoma cell growth although the quality of cross-priming, antigen presentation and immune cell recruitment within the tumor bed remain critical factors during the melanoma progression [33]. This has been proven either in murine melanoma models undergoing a rapid increase of tumor burden once depleted of CD8^+^ cells, or in patients whose treatment response is directly correlated to the density of TILs nearby the tumor cells [34–37].

The tumor immunity cycle is a process based on: a) a not-specific early phase driving the innate immunity that is mediated by macrophages, granulocytes, DCs and NK cells; b) late functional phase of effector CD4^+^ and CD8^+^ T-cells (T_effs_) primed against melanoma cells through the endogenous production of interferon-gamma (IFN-γ), a direct cytotoxicity tumor resulting from MHC-TCR interaction or the antigen-dependent activity of T-cells during the adaptive immune response [38].

*a) NK cells and DCs:* NK cells participate to the anti-melanoma immunity. In particular, they recognize and attack melanoma cells expressing low MHC class-I molecules with higher efficiency then T-cells [39] and promote functional interactions between the natural cytotoxicity receptors as NKG2D, NKp30, NKp44, NKp46 and relative ligands expressed by malignant cells [40]. Moreover, NK cells may indirectly contribute to immune-surveillance by enhancing the secretion of cytokines within the tumor microenvironment or by inducing the maturation of DCs thus concurring to the adaptive immune response [41]. In this context, it has been demonstrated that injection of IL-15-stimulated NK cells from murine melanoma may inhibit the tumor burden independently from cytotoxic CD8^+^ cells [42]. It has been also described that NK cells release perforins and granzymes within the tumor milieu in the presence of antigenic peptides that stimulate DCs and the T-cell cross-priming against the tumor cells [43, 44].

Mature DCs balance the efficiency of immune response and the ability of T-cells to orchestrate a cytotoxic effect. They physiologically circulate in peripheral blood and migrate to lymph nodes where they encounter naïve or memory T cells [45]. Mature DCs induce co-stimulation through CD40, CD80, CD86 and OX40L while they circulate in peripheral sites regulating innate and adaptive anti-melanoma immunity [46, 47]. The major mechanism required for DC maturation and efficient cross-priming include: i) the interplay of TCR with MHC molecules; ii) the binding of CD80/CD86 with CD28 expressed by T-cells; iii) the cytokine-mediated signals [48]; iv) the chemokine profile for migration from lymph nodes to distant tissues. To this regard, the melanoma milieu is enriched of immune suppressive cytokines as IL-6 and IL-10 as well as of miRNAs that propagate through the STAT-3 pathway [49] the survival of melanoma cells at expense of DCs [50, 51]. Lastly, it has been demonstrated that Th1 cytokines including IFN-γ and IL-12 directly activate both naïve and memory T-cells while providing the maintenance of anti-tumor CD8^+^ immunity and the modulation of T helper activity [52].

*b) Teff cells:* the efficiency of both CD4^+^ and CD8^+^ cells for the modulation of the adaptive immune response mostly depends on the specificity of the TCR signalling. T_effs_ play a pivotal role during the cell-mediated immunity through TCR-MHC interactions that is powered by IFN-γ and TNF-α. The primary role of adaptive immunity in melanoma is also addressed by the brisk T-cell infiltration that is considered a positive prognostic issue [53]. Moreover, CD4^+^ Teff cells commonly do not capture melanoma antigens from cells lacking MHC class-II molecules, although a number of studies proved that the majority of melanoma cells are restored in class-II expression by high levels of IFN-γ. The expansion of T lymphocytes activated against melanoma cells through the clonally distributed TCR, leads to the formation of elevated numbers of mRNA encoding the α and β chains of TCR. In particular, infiltrating T cells from murine melanoma models bear clonally expanded TCR transcripts whose activity is of great effort for the efficiency of the anti-melanoma immunity. In addition, several studies demonstrate that melanoma is characterized by a high number of clonally expanded T cells. However, the ‘selectivity’ of TILs is the consequence of the balance between clonality and specificity [54].

The general findings are that antitumor response may involve a variety of clonal TCRs that, notwithstanding a similar structure, may recognize the same HLA/peptide complex. Therefore, the relation between specificity and clonality has been clearly demonstrated proving that the clonally expanded cells within the tumor microenvironment are tumor-specific.

Finally, the attack of T-cells against melanoma cells results in their apoptosis by the release of perforin and granzymes followed by new antigen diffusion within the tumor bed. These sequential events allow the immunity cycle to start again and self-sustaining [55].

In conclusion, the results of this T-cell recruitment and activity within the tumor microenvironment predominantly include the induction of apoptosis in melanoma cells followed by the release of new antigens which once again allow to restart and self-maintaining of the anti-tumor immune cycle [56].

### Immune escape of melanoma

The escape of melanoma cells from the immune system control is regulated by the *‘immune editing’* that is mostly based on a complex machinery of intra- and extracellular signals. Immune editing is a dynamic process putatively involved in the colonization of distant tissues by melanoma cells and includes the ‘immune escape’ phase. It is driven by the chronic stimulation of the immune system and by strategies of malignant cells to counteract the immune-mediated antigenic recognition. Besides the progressive exhaustion of immune system to counterattack highly proliferating malignant cells, the escape also depends on the defective immune recognition as well as on the increased resistance to apoptosis of melanoma cells, or the development of an immunosuppressive microenvironment [57, 58].

The defective immune recognition is the first event useful to the melanoma cells for the immune escape and is due to inefficient antigen processing machinery [59] that progressively inhibits the ability of CD8^+^ T-cells to recognize the processed target antigens of the tumor cells [60]. Moreover, the effectiveness of T-cell cytotoxicity requires appropriate antigen presentation by mature DCs whose activity, co-stimulation and antigen presentation, critically induce a functional immunity. In this context, the maturation and priming of DCs is influenced by stimuli of the microenvironment where they suffer of an immature/tolerogenic phenotype induced by VEGF, IL-8 and IL-10 produced by melanoma cells. Moreover, the DC impairment [61] is also associated with reduced co-stimulation activity as result of the defective of CD80 and CD86 expression [62, 63].

Besides an intrinsic defect of DCs in their ability to arm the immune system against melanoma cells and the negative influence exerted by soluble factors produced by malignant cells, other populations including MDSCs [64, 65] and Tregs participate to the imbalance between immune suppression and tolerance. Recruitment and stimulation of MDSCs occur for the increased bioavailability of soluble factors as nitric oxide (NO), reactive oxygen species (ROS), TGF-Δ and arginase (ARG)-1 that are released by these cells and promote the inhibition of anti-tumor activity of T-cells and NK cells [40, 66]. In addition, Tregs are deregulated in melanoma and inhibit the immune system through TGF-Δ, IL-10 and IDO over-production which dampens the activity of CD4^+^ and CD8^+^ lymphocytes, and NK cells. Among these cytokines, IDO inhibits the effector T-cells by depleting tryptophan, while promotes the differentiation and activation of Foxp3^+^ Tregs by kynurenine production [67].

**Figure 1:**
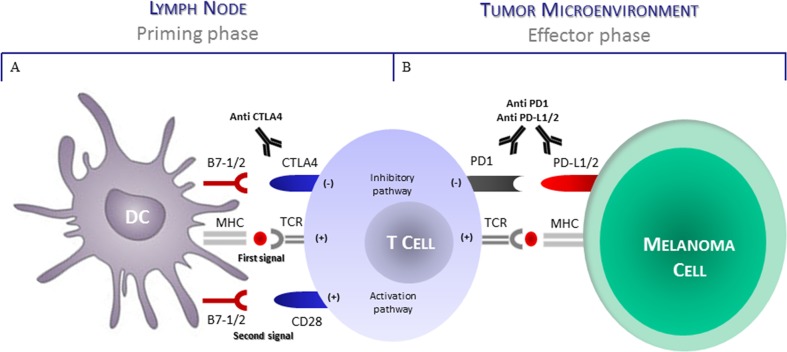
CTLA4 and PD1 regulate different stages of T-cell response **(A)** T cell activation requires two complementary signals. The interaction between TCR and peptide-MHC complex must be associated by a second co-stimulatory signal mediated by CD28. Conversely the binding of CTLA4 to B7-1/2 (CD80/86) provides a control signal that suppresses ongoing T-cell activation. **(B)** PD1 is upregulated on T cells after persistent antigen exposure. When PD1 binds its ligand as PDL1 and PDL2 expressed by tumor cells, the T cell receives on inhibitory signal. Antibodies direct to CTLA4 or PD1/PDL1 can activate T cells by preventing their functional disablement. *Abbreviations*: *DC*, dendritic cell; *CTLA4*, cytotoxic T lymphocyte antigen 4; *PD1*, programmed death 1; *TCR*, T-cell receptor; *MHC*, major histocompatibility complex; *PD-L1*, programmed death ligand 1; *PD-L2*, programmed death-ligand 2.

Another functional mechanism potentially affecting the anti-melanoma T-cell activity includes the defective expression of immune checkpoint receptors as well as their intrinsic binding capability by relative ligands [68]. The T-cells are functionally exhausted in relation to a decrease of cytokine production, as well as the ability to exert cytotoxicity [69]. Moreover, exhausted T-cells express inhibitory surface receptors as CTLA-4, PD-1, BTLA4, CD160, LAG-3, Tim-3 and VISTA [70–75] among these, CTLA-4 and PD-1 down-regulate the immune activity and are actually targeted to restore the anti-tumor immunity (Figure 1). A further inhibitory role exerted by these receptors include the reduction in the production of IL-2, IFN-γ and TNF-α as well as the transcription of intracellular signals leading to abnormal cyclin activity followed by the cell cycle arrest [76] (Table 1).

**Table 1:** Major mechanisms of immune escape in melanoma

*Mechanism*	*Population(s)/Pathway(s)*	*Effect(s) on TME*
Defective recognition of melanoma cells	Downregulation, mutation or loss of MHC class IMelanoma antigensDefective antigen presentation	Suboptimal activation of melanoma infiltrating lymphocytes
Negative feedback (up-regulation of the immune checkpoints)	CTLA4 PD1 LAG3 TIM3 VISTA	Inhibition of T cell function
Up-regulation of immune checkpoint ligands	PD-L1 PD-L2	Inhibition of T cell function
Up-regulation of immune suppressive populations	MDSCs Tregs	Inhibition of T cell functionDirect pro-tumorigenic effect (VEGF, TGF-beta)
Release of pro-apoptotic molecules by melanoma cells	FasL TRAIL	T cells death by apoptosis
Release of pro-tumorigenic andpro-angiogenic factors by TME	TGF-beta VEGF iNOS IDO IL-10 IL-6	Inhibition of T cell functionTumour angiogenesis and stroma remodeling

*TME*: tumor microenvironment; *MHC*: major histocompatibility complex; *CTLA4*: cytotoxic T-lymphocyte antigen 4; *PD1*: programmed death 1; *LAG3*: lymphocyte activation gene-3; *TIM3*: T cell immunoglobulin and mucin domain 3; *VISTA*: V-domain immunoglobulin suppressor of T-cell activation; *PD-L1*: programmed death ligand 1; *PD-L2*: programmed death ligand 2; *MDSCs*: myeloid-derived suppressor cells; *Tregs*: regulatory T cells; *FasL*: fas ligand; *TRAIL*: TNF-related apoptosis-inducing ligand; *TGF*: tumor necrosis factor; *VEGF*: vascular endothelial growth factor; *iNOS*: inducible nitric oxide synthase; *IDO*: indoleamine 2,3 dioxygenase; *IL*: interleukin.

### The effect of “targeted therapies” on the immune system in melanoma

The RAS/RAF/MEK/ERK cascade is the major deregulated pathway in malignant melanocytes. Mutations of *BRAF* are critical drivers of the melanoma proliferation in approximately 50-60% of cutaneous melanomas [77] whereas those of *NRAS* recur in 10-20% of patients [78] and are a negative prognostic factor. Treatment with BRAF/MEK inhibitors [79–82] is actually a cornerstone in melanoma although in some of them the duration response is limited and the best responders show peculiar clinical features [83].

Besides a specific anti-melanoma effect, however, targeted agents may potentially restore the immune system activity and early studies proved that inhibition of BRAF cascade raises the number of CD8^+^ T-cells nearby tumor cells and increases the exposition of TAAs synthesized by melanoma cells. Moreover, after exposure to BRAF inhibitors, a relationship between the number of infiltrating CD8^+^ cells and tumor burden following BRAF blockade has been described and the increased intratumoral CD8^+^ lymphocytes infiltration is apparently correlated with the reduction of the tumor size and enlarged necrosis in biopsies [84]. Furthermore, a reduced secretion of IL-10 and IL-6, and of VEGF by melanoma cells has been reported within the tumor milieu. A mechanism of resistance to targeted agents may include the high expression of TIM-3, PD-1 and PD-L1 by infiltrating T-cells in parallel with the increased production of IFN-γ in the tumor microenvironment [85]. On the contrary, the immune stimulation occurs during anti-BRAF treatment in consequence of the increased release of melanoma antigens [86] that are highly captured by T-cells regulated by mature DCs. Oncogenic BRAF contributes itself to the immune escape while inhibition of the MAPK pathway drives the expression of melanocyte differentiation antigens (MDA) by interfering with the transcriptional repression of micropthalmia-associated transcription factor (MITF) signature. It has been also demonstrated [87] a relationship between efficiency of MDA recognition by T-cells and benefit from immunotherapy and, contrariwise to previous studies suggesting a detrimental effect of MEK inhibitors, recent studies suggest that the immune response is preserved and potentiated by combinatory anti-BRAF agents [88]. Other potential mechanisms by which targeting BRAF might restore the immune system include the impairment of Tregs and MDSC activity [89] and the down-modulation of the C-C chemokine ligand (CCL)-2 that is primarily involved in melanoma cell recruitment within the tumor milieu [90].

**Figure 2:**
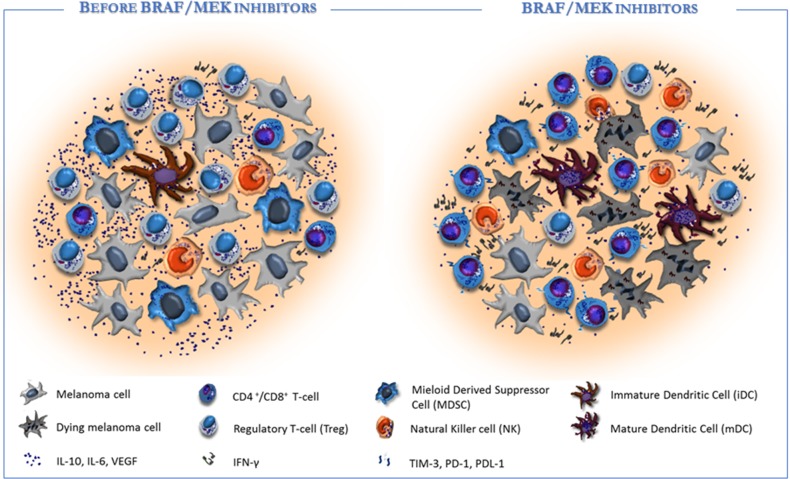
Targeted therapy affects the tumor microenvironment in favour of immune re-activation ***Left:*** Melanoma progression includes many pathogenetic and molecular events which contribute to the ineffective anti-tumor immunity. Melanoma microenvironment is enriched of immune-suppressive cytokines (e.g., IL-10, IL-6; TGF-β and VEGF) that drive the infiltration of immunosuppressive cells (e.g. Treg and MDSC), while impair the antigen processing machinery by DCs and the anti-tumor effect by T-cells and NK cells. ***Right:*** BRAF/MEK inhibitors exert direct anti-melanoma activity and restore the tumor immunogenicity within the microenvironment. Particularly, the targeted therapy induces the production of melanoma specific neo-antigens and hampers the immunosuppressive signals, thus restoring antigen presentation by DCs and T-cell mediated cytotoxicity. As a consequence, T-cells and NK cells increase nearby tumor, while Tregs and MDSC are strongly impaired. In addition, BRAF inhibitors may also condition tumor microenvironment in support of immunotherapy by inducing the expression of effector cell exhaustion molecules (e.g. PD-1 and TIM-3) on immune cells or PD-L1 on tumor cells.

The innate component of the immune system is also affected by targeted therapy and particularly the BRAF inhibitor PLX4720 increases the phosphorylation of ERK1/2, the CD69 levels, the IFN-γ secretion as well as either the proliferation or the cytotoxic activity *in vitro* and *in vivo* of NK cells [91, 92]. The dendritic cell function is also restrained by up-regulation of MAPK pathway in BRAF^V600E^ melanoma cell lines in terms of maturation, antigen capture, cross-priming and production of IL-12 and TNF-α [90, 93, 94]. By contrast, blockade of MAPK inhibits the negative effect of melanoma cells on the CD80, CD83 and CD86 expression thus restoring the DC co-stimulation (Figure 2).

These observations provide pre-clinical evidence supporting the use of combination MAPK inhibitors and immunotherapy in BRAF^V600^ mutated melanoma although the first clinical trial combining anti-CTLA4 and BRAF inhibitor resulted in highly severe hepatotoxicity [95]. Currently, studies investigating the safety and benefit of combinatory strategies in metastatic melanoma are in progress and include BRAF and MEK inhibitors, checkpoint blockers targeting CTLA4, PD-1 and PD-L1, NK-based immunotherapy and DCs vaccination.

## CONCLUSION

The concept that the immune system plays a critical role in controlling the tumor progression of melanoma is well established. Studies of the tumor microenvironment and the “immune contexture” [96, 97], understood as the presence of different immune variables associating the nature, density, functional orientation and distribution of immune cell populations, have provided knowledge on mechanism of the *immunosurveillance* and the immune escape. The understanding of these mechanisms has been crucial for the improvement of the treatment and for the establishment of new available immunotherapy strategies. Indeed, in the last few years, the immunotherapy showed the ability to obtain clinical and relevant benefit associated with durable responses. Besides the treatment of selected immunogenic tumors, the immunotherapy is currently considered an alternative strategy in other metastatic cancers, including urothelial, kidney, colorectal, head and neck as well as non-small cell lung cancers showing objective responses [30, 98–100]. However, many questions are still unanswered to obtain the optimization of immunotherapy.

The next objective in the treatment of this cancer includes the full recruitment of the immune system activity by targeting co-inhibitory and co-stimulatory molecules in sequence or in combination with the targeted therapies such as the BRAF/MEK inhibitors, the approaches to improve the function of innate immune response, the cytokines, the IDO inhibition, the adoptive cell transfer and T-cell engineering, the therapeutic vaccines in combination with ongoing therapeutic approaches (NCT02178722; NCT02327078; NCT01656642; NCT02130466; NCT02263508).

The research of soluble and local immunologic biomarkers or the corresponding molecular profile of melanoma could also provide the valuable prognostic and predictive knowledge for the best therapeutic decision and identify novel targets for immunotherapy. However, melanoma remains a “model cancer” to increase our understanding of the immune-oncology to be also extended to other tumor types.
